# CO and O_2_ Interaction with Kinked Pt Surfaces

**DOI:** 10.1021/acscatal.4c00435

**Published:** 2024-04-10

**Authors:** Fernando García-Martínez, Elia Turco, Frederik Schiller, J. Enrique Ortega

**Affiliations:** † Centro de Física de Materiales CSIC/UPV-EHU-Materials Physics Center, Manuel Lardizábal 5, San Sebastián 20018, Spain; ‡ Departamento Física Aplicada, Universidad del País Vasco, San Sebastián 20018, Spain; § Donostia International Physics Centre, Manuel Lardizábal 4, San Sebastián 20018, Spain

**Keywords:** CO adsorption, O_2_ adsorption, platinum, curved crystal, kinked surface, X-ray photoemission spectroscopy

## Abstract

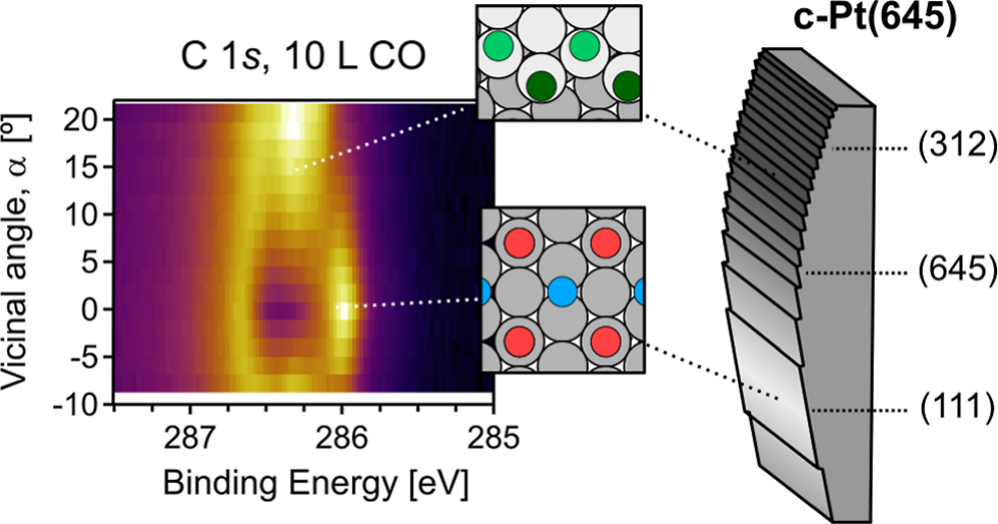

We investigate the chemical interaction of carbon monoxide (CO) and oxygen (O_2_) with kink atoms on steps of platinum crystal surfaces using a specially designed Pt curved sample. We aim at describing the fundamental stages of the CO oxidation reaction, i.e., CO-covered/poisoned stage and O-covered/active stage, at the poorly known kinked Pt facets by probing CO uptake/saturation and O_2_ saturation, respectively. Based on the systematic analysis that the curved surface allows, and using high-resolution X-ray photoemission, a diversity of terrace and step/kink species are straightforwardly identified and accurately quantified, defining a smooth structural and chemical variation across different crystal planes. In the CO-saturated case, we observe a preferential adsorption at step edges, where the CO coverage reaches a CO molecule per step Pt atom, significantly higher than their close-packed analogous steps with straight terrace termination. For the O-saturated surface, a significantly higher O coverage is observed in kinked planes compared to the Pt(111) surface. While the strong adsorption of CO at the kinked edges points toward a higher ignition temperature of the CO oxidation at kinks as compared to terraces, the large O coverage at steps may lead to an increased reactivity of kinked surfaces during the active stage of the CO oxidation.

## Introduction

The study of the carbon monoxide (CO) oxidation stands as fundamental in comprehending surface catalytic reactions, holding a prominent place in surface science research.^1−4^ However, most investigations to date have occurred under controlled conditions, either using single crystals or in ultrahigh vacuum (UHV) environments, detached from the real conditions of industrial applications. Consequently, extending surface science findings to real catalytic systems, operating at atmospheric pressures (pressure gap), and involving powder/nanoparticle catalysts (materials gap), may lead to inaccuracies.^5^ New experimental approaches are needed to allow the most powerful techniques to bridge both the pressure gap, such as near ambient pressure x-ray photoemission (NAP-XPS), and the materials gap through novel sample designs. In the latter case, it is important to realize that metallic nanoparticles possess multiple facets, making it difficult to track their specific activity and interactions during the chemical reaction.^6^ Therefore, conventional single-crystal surfaces offer limited information as they represent only one plane, failing to mirror the complex, multifaceted structure of actual catalysts.^7^ One potential approach to bridge the structural gap involves the utilization of cylindrical sectors of single crystals. Their curved surfaces allow for a smooth transition in the crystal orientation, enabling a systematic comparison of various facets under identical reaction conditions. Moreover, with a proper selection of the crystal sector, a curved surface also offers a sophisticated but consistent means of analyzing vicinal surfaces, hence the effect of undercoordinated, step atoms in surface-catalyzed reactions.^8−11^

**Figure 1:**
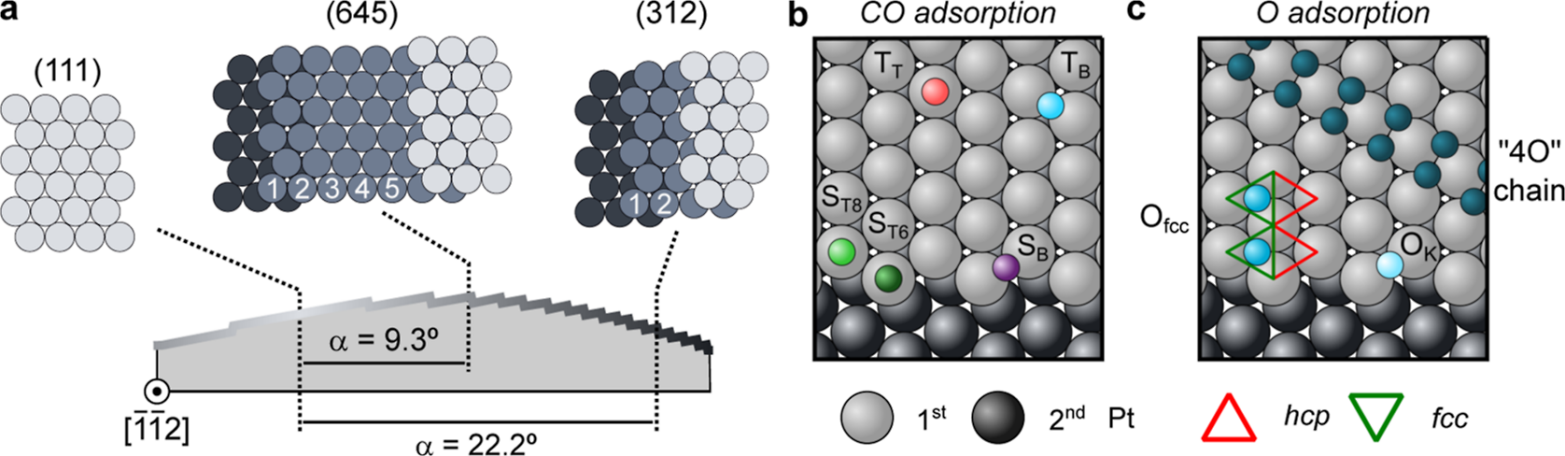
c-Pt(645) sample. (a) Side-view description of the sample, which is a cylindrical Pt crystal sector, with cylinder axis parallel to the [112̅] direction. The atomic models sketch the characteristic (645) and (312) planes, featuring kinked steps and (111) terraces of 5 and 2-atom-rowwidth, respectively. In (b,c), we sketch relevant adsorption geometries for CO and O at (111) terraces and stepped kinks. For CO, T_T_ and T_B_ stand for molecules adsorbed in Terrace-Top and Bridge positions, while S_T6_, S_T8_, and S_B_, respectively, refer to the CO adsorbed on Step-Top sites in the first (six-fold-coordinated Pt), the second (eight-fold-coordinated Pt) row, and at Step-Bridge positions. For oxygen, O_fcc_ and O_K_ correspond to O adsorbed at three-fold *fcc* hollow and bridge kink-sites, respectively, while “4O” refers to oxygen arranged in a local Pt chain structure with PtO_4_ motifs (see the main text).

The combined use of curved surfaces with (NAP-)XPS has successfully demonstrated its potential to straightforwardly assess the role of steps in the CO oxidation on Pd, Pt, and Rh vicinals^12−14^ or Ag-oxidation,^15^ among other matters.^16^ The curved geometry allowed the identification of surface species at different reaction stages and an accurate determination of the ignition temperature across different facets. Surprisingly, species and ignition temperatures were found the same at A-type ({100}-oriented microfacets) and B-type ({111} microfacets) stepped Pt(111) surfaces,^14^ by contrast to the expected A/B asymmetries observed in Pd and Rh.^12,13^ Yet the question arises whether such homogeneous and symmetric behavior in Pt occurs far beyond the (111) plane or features different step geometries in the vicinity of the Pt(111) surface, such as the more open kinked steps (Figure 1a). Their complexity is probably the reason why they have been poorly investigated with XPS in the CO oxidation context, even under UHV conditions.^17,18^ Note that the UHV-XPS study is a very relevant step^8,10,11^ since it allows to determine core-level shifts for the variety of chemical species that arise in the presence of steps, thereby providing accurate reference spectra for NAP-XPS, which generally exhibits lower resolution and poorer statistics.

CO and O_2_ adsorption studies using UHV-XPS are abundant for Pt(111) and its A/B vicinal surfaces; hence, the resulting CO/Pt and O/Pt interfaces and adsorption sites are well established. They are sketched in Figure 1b,c. Exposing the (111) plane to CO at room temperature (300 K) will typically give rise to a *c*(4 × 2)-4CO pattern with 0.5 monolayers (ML, one adsorbed molecule per substrate surface atom),^19^ while a more dense overlayer packing (0.68 ML) can be achieved when exposing the surface to CO in NAP conditions.^20−22^ In Pt(111) close-packed vicinals, CO is known to adsorb on top sites at the upper edge (S_T_) of B-type steps.^23−25^ In A-type steps, in addition to the S_T_ occupation, a small amount of CO adsorbs at bridge positions (S_B_).^26−29^ On the other hand, exposing the Pt(111) surface to O_2_ above 77 K leads to the immediate molecular dissociation, with O atoms arranged in a *p*(2 × 2) layer.^30−32^ This structure corresponds to 0.25 ML coverage with O atoms occupying three-fold *fcc* hollow sites.^33−35^ Higher O coverages lead to the formation of one-dimensional (1D) oxygen stripes, eventually condensing into a structure where rows of Pt surface atoms appear linked to four oxygen atoms each (“4O” Pt surface oxide).^36−39^ At Pt-stepped surfaces with (111) terraces and A/B-type steps, the dissociative O_2_ adsorption and the subsequent chemisorption are favored at steps over terraces,^40−44^ resulting into oxygen atoms anchored to bridge sites at A-type steps, and hollow *fcc* positions close to the edge at B-type steps.^41,45−47^ Finally, after the saturation of the oxygen chemisorption, 1D “4O”-like chains along the step edges were also observed for Pt vicinal surfaces with A/B steps.^40,48,49^ Adsorption experiments on vicinal Pt(111) surfaces with kinked steps are much scarce. Early works on the CO adsorption on kinked Pt surfaces reported S_T_ as the preferential site, although a small amount of S_B_ may also be present.^17^ For O_2_, while it is clear that O binds stronger on the kinks as compared to terraces, the nature of the adsorbed species remains unknown.^50−52^ An XPS study by Held et al. in Pt(531)^18^ reported three different species after annealing the surface in ≈10^–7^ mbar O_2_, i.e., one chemisorbed state and two types of oxide clusters.

Using a Pt curved crystal with a special design and UHV-XPS, here we investigate a wide range of Pt vicinal surfaces with kinked steps during CO and O saturation at 300 K. They represent the two fundamental stages of the CO oxidation reaction, i.e., the CO-poisoned and the O-active surfaces. The sample is a cylindrical sector of a Pt crystal, with cylinder axis parallel to the [112̅] direction (see Figure 1 and Methods in the Supporting Information) and the (645) direction at the center of the curved surface [c-Pt(645) sample]. The reference (111) plane is located close to one edge, allowing Pt vicinal surfaces to be spanned with kinked steps up to a α = 28° vicinal (or tilt) angle with respect to the (111) plane. The large vicinal angle range allows us to reach all vicinal surfaces from the (111) beyond the densely kinked (312) surface (2-atom-wide terraces) at the opposite sample edge. Through uptake and saturation experiments on this sample, we identify a variety of CO and O species, which can also be quantified and discussed in model adsorbate structures, thanks to the systematic approach that only the curved geometry allows.

## Experimental Methods

### Sample Cleaning and Characterization

The c-Pt(645) sample, featuring the (645) plane at its apex, is described in Figure 1. The sample is cleaned with Ar^+^ sputtering cycles at 1 kV, O_2_ annealing (1 × 10^–7^ mbar, 800 K), and final flashes (950 K) until no contaminants are seen in the XPS spectrum. Low-energy electron diffraction (LEED) was employed to probe the surface structure of the curved surface. Diffraction patterns and the Pt 4*f* XPS region of the clean c-Pt(645) crystal are shown in Figure S1.

### X-ray Photoemission Experiments and Peak Fitting

XPS was carried out at the SuperESCA beamline of Elettra synchrotron in Trieste (Italy)^53^ in normal emission geometry. The beam fingerprint is ≈20 μm in the vertical direction, i.e., along the perpendicular direction of the steps and the curvature of the crystal, allowing probe of vicinal surfaces within an accuracy below 0.1°. We observed beam damage effects during a long measurement (>30 min) in Pt(111) after CO saturation, as a small fraction of CO adsorbed at T_B_ dissociated toward “C”. Therefore, we reduced our measurement time at the positions of the α-scan (<10 min) to minimize beam damage effects. The peak fitting was performed using the *lmfit* package of Python.^54^ Doniach–Sunjic line shapes^55^ convoluted with a Gaussian were considered for the asymmetric peaks (adsorbed CO in the C 1s, graphitic C, and atomic O), while Voigt profiles were used for symmetric peaks (adsorbed CO in the O 1s, “4O”-like stripes), together with a Shirley-type background.^56^ Small peaks ascribed to vibrational excitations of CO molecules^57−59^ could be distinguished in the high-resolution C 1s spectra of Figure 3; hence, they were included in the fitting procedure. From the fit of the (111) surface, the satellites were fixed at 220 meV toward higher binding energy of the main contribution, and the intensity ratio derived was 11.5% and 10% for T_T_ and T_B_ sites, respectively. The other parameters were constraint to those of the major peak, and their area was added to that of the main feature for quantification. The intensity ratio of the T_T_ satellite was applied for the S_T6_ and S_T8_ satellites. In the case of the uptake and desorption experiments, spectra were acquired considerably faster than the high-resolution spectra. For this reason, S_T6_ + S_T8_ were not resolved; hence, a single peak S_T_ was employed. Satellites were neither resolved.

### Coverage Calibration

The coverage of high-resolution spectra of Figure 3 was calibrated using the *c*(4 × 2) pattern observed by LEED as a reference. As described in ref.,^25,29,60^ a factor extracted from the (111) needs to be applied to CO adsorbed in bridge sites to properly account for their coverage. Such a factor was applied to the intensity of T_B_ when converting peak area to coverage. In uptake experiments, coverage was calculated by assuming saturation of T_T_ sites in the (111) plane measurement. In the case of the desorption experiments, since the photon energy is different, we also assumed CO saturation at the beginning of the desorption ramp at the (111) surface. O 1s intensities were calibrated using the *p*(2 × 2)-O superstructure observed in the (111) plane by LEED.

## Results

### CO Uptake

**Figure 2:**
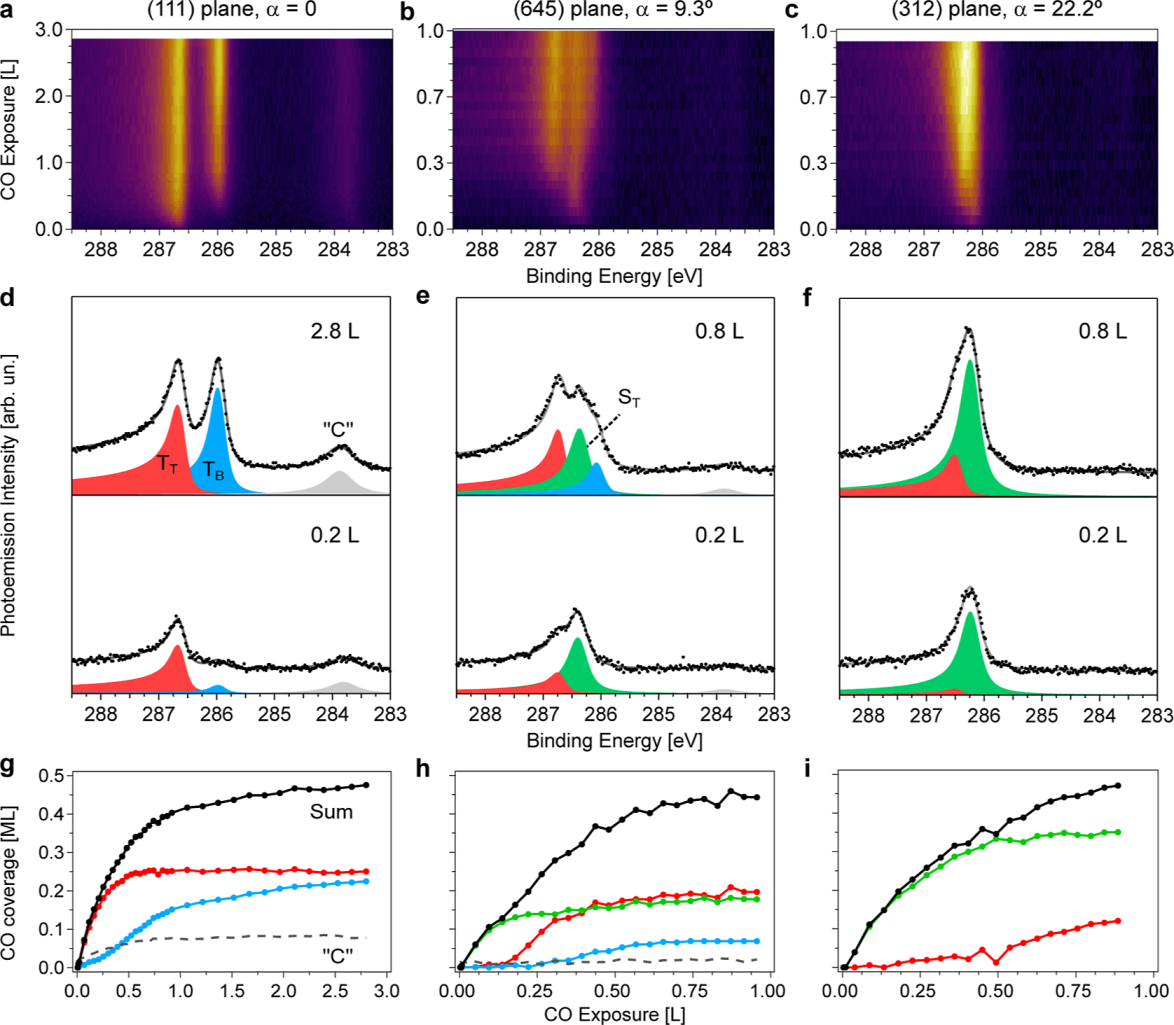
CO uptake at the (111), (645), and (312) Pt surfaces. (a–c) C 1s color plots acquired during CO dosing at the (111), (645), and (312) surfaces on the c-Pt(645) sample. Experiments on the (645) and (312) surfaces were carried out at constant CO pressure of 1 × 10^–9^ mbar, whereas for the (111) plane, a pressure increase to 1 × 10^–8^ mbar CO was required to speed up the process. (d–f) Characteristic spectra and fitting lines at low (top) and high (bottom) CO exposures for each plane. (g–i) Coverage evolution of each CO species as a function of CO exposure. T_T_, T_B_, and S_T_ stand for CO adsorbed as Terrace-Top, Terrace-Bridge, and Step-Top sites, as sketched in Figure 1. The dashed black lines represent the graphitic carbon (“C”) contribution, while the solid black line corresponds to the total CO coverage. The measurements were performed at a photon energy of 370 eV and 300 K.

In order to identify preferential adsorption sites, separate CO uptake experiments were performed at the (111), (645), and (312) surfaces on the c-Pt(645) sample. These surfaces were exposed to 1 × 10^–9^ mbar of CO at 300 K while continuously recording the C 1s photoemission peak, the latter shown as color plots in Figure 2a–c. In Figures 2d–f, we display individual spectra at 0.2 L and close to saturation, together with their respective peak fit analyses, from which peak intensities of each core-level line are determined. The latter and the total C 1s intensity are represented in Figure 2g–i for the three different surfaces. As shown in Figure 2d, in the (111) surface, and shortly after the CO exposure begins, a peak appears at 286.7 eV, which belongs to CO anchored atop Pt(111) atoms (Terrace-Top, T_T_).^60^ Later, and at a slower pace, another feature develops at 286.0 eV, which corresponds to CO adsorbed at bridge sites (Terrace-Bridge, T_B_).^31,60,61^ The evolution of these two CO species is shown in Figure 2g. While the T_T_-CO intensity steadily grows until saturation at around 0.6 L, the adsorption at the T_B_ sites is slower and approaches saturation after the highest dose used here (3 L of CO). Close to surface saturation, the ratio T_T_/T_B_ is close to 1. This is expected from the *c*(4 × 2) superstructure and agrees well with a preceding report on the CO adsorption on Pt(111) at 200 K.^60^ Finally, we note the presence of a sizable amount of graphitic carbon (“C”) in Figure 2d, which becomes residual in the (645) spectrum in Figure 2e, and completely disappears in the (312) case in Figure 2f. This points to CO terrace species as particularly sensitive to beam-induced dissociation. In fact, exposing the CO-saturated (111) surface to the beam for approximately 30 min reveals a slow preferential dissociation of CO-T_B_, since CO-T_T_ remains constant as “C” increases and T_B_ decreases with beam exposure (not shown).

The center column of Figure 2 illustrates the uptake experiment for the kinked (645) plane (α = 9.3°, 5-atom-wide terraces). As seen in Figure 2e, a peak at 286.4 eV emerges prior to any other feature. Similar to Pt vicinals with A- and B-type steps,^25,29^ and in agreement with earlier reports on kinked Pt surfaces,^17^ we attribute this peak to CO adsorbed in top positions at the kinked edge (Step-Top, S_T_). At higher CO exposures, adsorption at the terrace T_T_ and T_B_ sites is observed. The coverage evolution of Figure 2h shows how adsorption at S_T_ sites slows down at around 0.15 L of CO, the point at which adsorption at T_T_ sites starts. As in the Pt(111) surface uptake, the occupation of T_T_ positions is followed closely by that at the T_B_ sites. T_T_-CO grows faster than T_B_-CO and stops at around 0.5 L of CO. However, T_B_-CO continues to increase at a quite reduced rate up to 0.7 L of CO. At the highest exposure of this experiment (1 L of CO), the surface is not saturated yet.

The CO adsorption kinetics on the (312) surface (α = 22.2°, 2-atom-wide terraces) is depicted in the right column of Figure 2. Again, S_T_-CO arises immediately after introducing CO to the chamber, followed by adsorption at T_T_ sites. As seen in the coverage evolution (Figure 2i), S_T_-CO saturates at around 0.6 L of CO, in contrast to T_T_-CO, which does not yet saturate at 1 L of CO. The increase in the saturation dose of CO adsorbed at S_T_ sites is simply explained by its higher step density as compared to the (645) plane. At the same time, the effective terrace length of the (312) surface is strongly reduced with respect to the (645) plane. Accordingly, the coverage of T_T_-CO and T_B_-CO decreases from the (645) plane to the (312) plane. At the latter, the coverage of T_T_-CO is very small, and T_B_ is not even observed. This agrees with early studies on the CO adsorption by electron energy-loss spectroscopy on the very same surface^17^ and by XPS on the fully kinked Pt(201) surface plane (α = 39.2°, 1-atom-wide terraces).^62^

A joint analysis of the three uptake experiments allows us to establish the CO adsorption sequence as S_T_ → T_T_ → T_B_, whereas the CO desorption experiments shown in Figures S2 and S3 of the Supporting Information confirm the expected reverse sequence of desorbing species, i.e., T_B_ → T_T_ → S_T_. Therefore, the impinging CO molecules adsorb first on kinks in the low coverage regime, and they desorb from kinks only after the CO from the terraces has almost vanished. Such sequence is of importance for the CO oxidation reaction as its activation is triggered by the desorption of CO. In fact, a slightly larger activity of the (111) facet compared to other surfaces with A- and B-steps was observed before the reaction light-off.^14^ Furthermore, Pt(111) was found to ignite earlier than Pt(557).^63^ As described by Rempel et al. for the case of the NO reduction,^64^ and following Sabatier’s principle, a strong adsorption of reactants may poison a surface reaction. Therefore, operando studies are needed to address the activation of the CO oxidation in densely kinked surfaces, such as (312) studied in this work.

### C 1s α-Scan at CO Saturation

**Figure 3:**
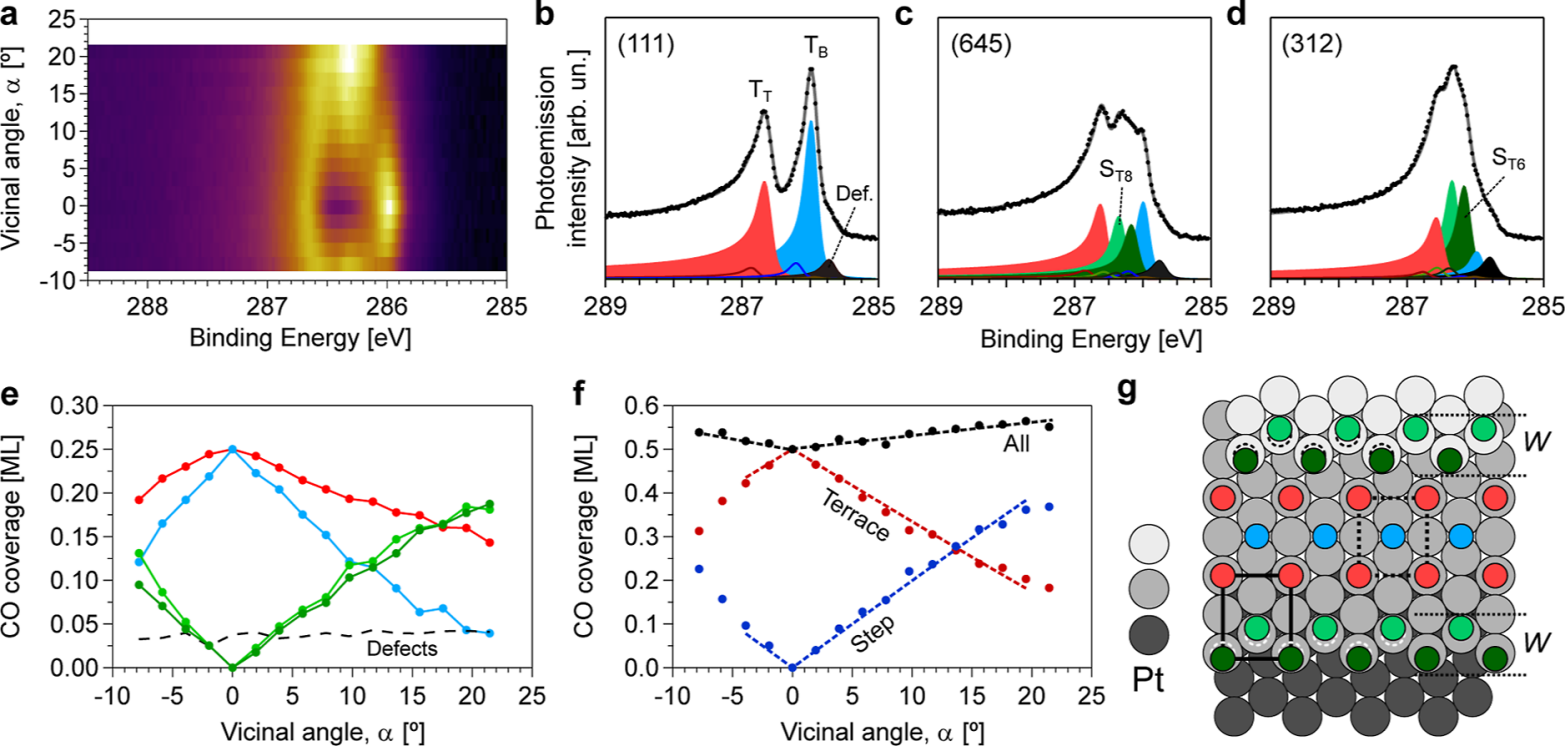
C 1s α-scan at CO saturation. (a) C 1s intensity color plot across the curved c-Pt(645) crystal after exposure to 10 L CO at 300 K. (b–d) Selected spectra at the (111), (645), and (312) surfaces, respectively, together with their corresponding fits. For each species (T_T_, T_B_, S_T6_, and S_T8_), higher binding energy satellites are added to account for vibrational losses. The small low-binding energy peak (black filling) peak has a constant intensity across the curved crystal and is assigned to CO adsorbed at defects. Photoemission spectra were recorded at 90 K and at a photon energy of 370 eV. (e,f) CO coverage variation of individual and total CO species as a function of α, extracted from the fit of individual spectra of the α-scan in (a). A small amount of “C” around 284 eV, similar to that of the defects, was also observed. (g) CO layer structure at the (645) plane, as derived from the data analysis. At steps, a slight shift from pure atop sites is suggested, in order to minimize repulsion. Lines in (f) are fits to data using the *W*-model that renders the effective size of the kinked steps *W*, as indicated in (g).

To fully characterize the CO species across the entire curved surface, we move the sample relative to the X-ray light spot in Δ*α* ≈ 2° steps and after saturating the surface with a 10 L of CO exposure at 300 K. Figure 3a shows the resulting C 1s intensity map, called α-scan, acquired at 90 K to improve the energy resolution. The C 1s α-scan allows us to clearly visualize the vicinal-angle-variation of species deduced from the uptake and desorption experiments at selected angles in Figure 2. The curved surface was also probed by LEED after CO saturation, as shown in Figure S1 of the Supporting Information. At the (111) plane, one obtains a sharp *c*(4 × 2) pattern that corresponds to the 0.5 ML CO saturation coverage at 300 K.^19^ Therefore, we can use the C 1s spectrum in the (111) surface to calibrate the CO coverage across the entire curved sample. The corresponding O 1s data for the 10 L CO dose, as well as for a lower dose of 0.25 L, can be found in Figure S4 of the Supporting Information. At the (111) surface, peaks coming from T_T_ and T_B_ are resolved at 532.5 and 531.0 eV, respectively. However, since the S_T_ line is too close to the T_T_ peak, these are very difficult to resolve.^25^ We therefore resorted to the C 1s region in order to study individual CO species on kinked surfaces.

Individual C 1s spectra corresponding to the (111), (645), and (312) surfaces are depicted in Figure 3b–d. At the (111) plane, T_T_ and T_B_ components are neatly observed, as well as the small shoulders ascribed to vibrational excitations of CO.^57−59^ At the (111) surface, the ratio T_T_/T_B_ is close to 1, as expected from the *c*(4 × 2) superstructure.^19^ In the (645) and (312) planes, the improved resolution allows us to unveil a double structure in the kink-related peak, which we ascribe to CO adsorption on top of six-fold-coordinated Pt atoms at the kink edge (S_T6_, 286.17 eV) and at the eight-fold-coordinated Pt atoms of the second row (S_T8_, 286.34 eV), both sketched in Figures 1c and 3g. S_T8_, S_T6_, and T_B_ peaks exhibit almost constant binding energy all over the α-scan, whereas T_T_ smoothly shifts from 286.67 eV at the (111) plane to 286.56 eV at the (312) surface, likely reflecting the change in the average strain of Pt atoms within (111) terraces as they get narrower.^8^ Finally, the small low-binding energy feature at 285.8 eV has a similar energy and intensity in all three spectra and all across the α-scan. Such lack of dependence on the step-density is the main reason to believe that this peak is related to CO adsorbed at defects,^29^ discarding CO anchored at terrace-hollow or step-bridge sites, with similar binding energy.^25,29,65^

The coverage evolution of individual CO species as a function of α at saturation is shown in Figure 3e. The shown data are the peak integrals determined from the peak fits performed on individual spectra from the α scan. The linear variation is characteristic of the curved surface,^10,13^ reflecting the fact that the number of step sites increase and terrace sites decrease linearly as a function of α. The intensities from the kink-CO peaks S_T8_ and S_T6_ rise with α at an almost identical rate, indicating a similar occupation of the two sites at the kinked edge, irrespective of the step density. In contrast, T_T_- and T_B_-CO decrease at different pace. This behavior is also found in close-packed A-type vicinal surfaces^14^ and will be discussed below.

From the coverages derived from the α-scan, we may estimate the effective size of terraces and kinked steps, i.e., the portion of the surface that is covered by terrace-like (T_T_, T_B_) and step-like (S_T6_, S_T8_) CO species, using the so-called *W*-model (see ref (10) and the Supporting Information for a full description and detailed analysis). In the *W*-model, all step species are confined within a stripe of width *W* around step edges, which remains constant as the step density increases. As a consequence, the intensity of step-like species is expected to increase linearly as a function of α, while terrace-like species decrease with it as the terraces narrow. Therefore, for the *W* model, we will consider the total step (S_T6_ + S_T8_) and terrace (T_T_ + T_B_) contributions, represented in Figure 3f, and fit their linear variation, using Θ_T_^0^ = 0.5 ML, the saturation coverage at the (111) surface, as a single fitting parameter. The fit returns the effective step size *W* = 4.3 ± 0.1 Å, sketched in Figure 3g, as well as Θ_S_^0^ = 0.71 ± 0.02 ML, i.e., the local coverage within the *W*-region. From the latter, we straightforwardly deduce that approximately one CO molecule adsorbs per kink atom (see the Supporting Information), i.e., there is a full CO occupation of the kinked edge at all vicinal angles. Full occupation at 300 K is also found in, e.g., Ni(100) vicinals,^66^ and also in the close-packed Pt(332) and Pt(557) surfaces but with CO pressures in the mbar range.^67^ We conclude that kinked steps may accommodate a significantly higher amount of CO as compared to close-packed steps at a reduced pressure. Due to the high CO-coverage at the upper kink edge, one could expect a large increase of the total CO coverage with the vicinal angle α. However, the understep region remains CO-depleted, which compensates for the high CO coverage at kinks, resulting in and leads to a slight increase of the total CO coverage with α.

The CO-covered vicinal planes across the c-Pt(645) sample show no LEED spots other than those arising from ordered monatomic step arrays, similar to B-type but contrary to A-type Pt vicinals.^29^ However, with the information extracted from the evolution of individual species of Figure 3e and the *W*-model, we may still postulate the structural adsorption model for the (645) plane sketched in Figure 3g. Pt rows are sequentially filled by CO molecules from the upper kink edge and toward the inner terrace, first in T_T_ sites and next in T_B_ positions, developing a *c*(4 × 2)-like superstructure. Therefore, CO anchoring at T_T_ is favored over that at T_B_, hence explaining the faster decay of T_B_ with α observed in Figure 3e. In reality, the sequential filling from the step edge and inside the same terrace proposed in Figure 3g does not change the *c*(4 × 2)-4CO density at each terrace, although it may slightly increase the total α-dependent coverage due to the proximity of the first occupied T_T_ site in the lower terrace. This small coverage increase is indeed detected in the total intensity curve of Figure 3f. Finally, both Θ_T_(α) and Θ_S_(α) deviate from linearity above α > 18° in Figure 3e. This indicates that local coverages, hence adsorbate structures around steps and terraces, vary at densely kinked surfaces that feature narrow terraces, such as the (312) plane.

### O 1s α-Scan at O Saturation

**Figure 4:**
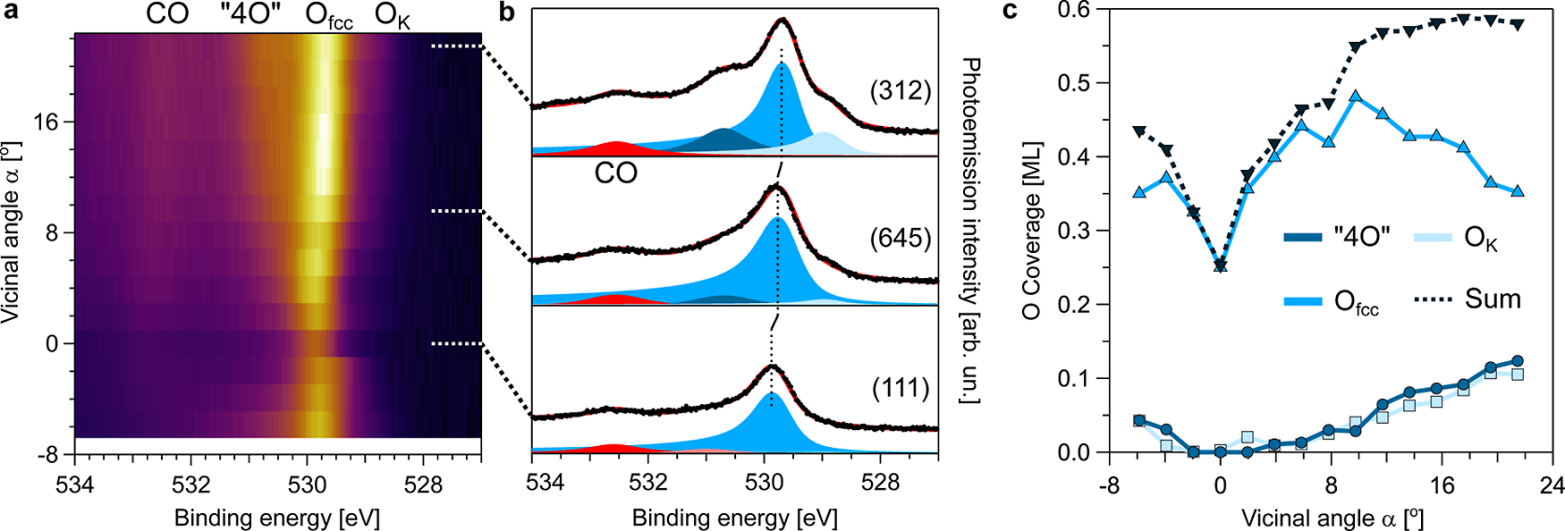
O 1s α-scan saturation of the c-Pt(645) surface. (a) O 1s α-scan acquired after O_2_ dosing of 8 L at 300 K and subsequent cooling to 90 K. (b) Individual spectra at the (111), (645), and (312) surfaces with line fitting. O_fcc_, “4O”, and O_K_ stand for chemisorbed O at *fcc* hollow sites, the “4O”-like 1D stripes along the step edges, and chemisorbed O at bridge sites of kinked edges (see the text and Figure 1). The high binding energy features (red filled peaks) correspond to residual CO, built up over the measuring time. (c) Coverage variation as a function of α for separate O species and their sum, as extracted from the fit to all individual spectra in the α-scan. Experiments were carried out at a photon energy of 650 eV.

After the ignition of the CO oxidation in Pt-like metals, the surface of the catalyst is typically covered by oxygen species that are reactive toward CO_2_ production.^3^ To characterize the O layer in the Pt-kinked planes, we saturate the c-Pt(645) sample with a 8 L O_2_ dose at 300 K. The corresponding α-scan is shown in Figure 4a, and selected O 1s spectra at relevant surfaces appear in Figure 4b. Measurements were performed at 90 K, which led to a small buildup of CO from the residual gas during the acquisition of the spectra. At such temperatures, CO does not react with the oxygen adsorbed on Pt but rather sticks to the surface.^68^ However, if a similar experiment is performed at 300 K (Figure S5 of the Supporting Information), an O coverage reduction is observed as compared to the results acquired at 90 K as the residual CO removes some of the oxygen atoms from the surface by forming CO_2_.

The O 1s spectrum of the (111) plane contains a single, asymmetric peak at 529.7 eV, which corresponds to chemisorbed atomic O in *fcc* hollow sites (O_fcc_).^68^ As confirmed by LEED (not shown), O atoms arrange in a 0.25 ML, *p*(2 × 2)-O structure at the Pt(111) surface.^32,69^ Therefore, the 1s intensity of the O at this crystal position is used for coverage calibration. At the (645) surface, the intensity of O_fcc_ shifts from 529.7 to 529.6 eV and reaches its maximum intensity. The binding energy position keeps decreasing with α up to the (312) plane, exhibiting at the same time an intermediate intensity between those achieved at the (645) and (111) facets. This suggests that O atoms anchored at three-fold-coordinated surface *fcc* sites in both step edges and terraces (see Figure 1c) contribute to the O_fcc_ peak, with its binding energy shift reflecting the step/terrace occupation ratio. In fact, our data agrees with the O_fcc_ intensity increase and peak shift observed between Pt(111) and Pt(332),^40^ and also with earlier studies reporting the adsorption of O at *fcc* sites in B-steps.^41,45−47^

In addition to the presence of O_fcc_, two small contributions appear at 528.8 and 530.7 eV in the (645) plane, both growing in intensity in the densely stepped (312) plane. Therefore, these features correspond to different oxygen species anchored at steps. The lower binding energy peak at 528.8 eV points to the presence of chemisorbed oxygen. No similar feature was observed in UHV conditions for the Pt(332) and Pt(553) surfaces with close-packed B-steps,^14,40,48^ although it was detected for the Pt(223) facet with square {100} microfacets.^14,48^ Since oxygen is reported to adsorb at bridge sites at {100} steps,^41,45−47^ we therefore ascribe this peak to O chemisorbed at the bridge sites of the {100} microfacet in the kinked step (O_K_). However, O_K_ exhibits a shift toward lower binding energy as compared to O_fcc_. This would be indicative of an increase in the coordination number of the chemisorbed oxygen,^70^ resulting into adsorption at the square sites of the square A-step instead of bridge sites. We cannot confirm this scenario with our experimental data set and follow the assignation of O adsorbed at bridge sites. However, given the proximity between square and bridge sites at the {100} microfacets, we believe that O_K_ is likely displaced from a pure bridge geometry toward the position of the square site, in a bridge-like configuration. On the other hand, the 530.7 eV emission is assigned to oxygen sitting in 1D “4O”-like stripes along the step edges.^40,48,49^ Such O site occupations occur right after the chemisorption process.^40,48,49^ The small amount of “4O” implies that only a small fraction of the Pt kinked surfaces develops 1D “4O”-like chains across the kinked edge, in agreement with observations in Pt(332),^40^ Pt(553),^48^ and Pt(533).^49^ The energy of “4O” is close to that of adsorbed OH.^71^ However, a peak similar to “4O” was observed during CO oxidation around 1 mbar in close-packed Pt vicinal surfaces^14^ and in the kinked Pt(531) after annealing in O_2_ in UHV.^18^ Under such conditions, the presence of OH groups is unlikely. Nevertheless, we do not discard a small contribution from OH groups to the “4O” intensity, particularly at 90 K. Both the values of the O_K_ and “4O” are sketched in Figure 1.

To gain more quantitative insights into the different adsorbed species, we fit all individual spectra in the α-scan of Figure 4a. The α-dependent O 1s intensity (expressed in MLs) obtained for each peak and the total O coverage is shown in Figure 4c. As expected for step-like species, the coverage of both the O_K_ and the “4O”-like species grows linearly as a function of |α|. In contrast, O_fcc_ does not exhibit any α-dependent terrace-like decreasing trend but reveals a sharp coverage increase from 0.25 to 0.35 ML in the α = 0–10° range, followed by a smooth decrease above α = 10°. Such nonlinear, α-dependent occupation of O_fcc_ sites reflects the double step-terrace contribution to the O_fcc_ peak discussed above. It is interesting to note that the steep increase in the intensity of the O_fcc_ peak goes in parallel with the rapid attenuation of the LEED pattern away from the (111) direction. The latter becomes fuzzy and shows no signature of step-ordering in vicinal planes. This suggests that, in the presence of O atoms, kinked step edges roughen,^72^ allowing a concentration of O_fcc_ species (steps + terraces), higher than 0.25 ML. The total O coverage (O_fcc_ + “4O” + O_K_) almost doubles from the (111) plane (0.25 ML) to the (312) edge of the sample (0.46 ML) at α = 22°. It is in fact expected that the oxygen coverage of open stepped surfaces is larger as compared to the (111) plane upon similar treatments,^18,40,44,48,49,73^ reflecting the higher reactivity of steps. Finally, the oxygen species observed for Pt(332)^40^ and Pt(531)^18^ were reported to be significantly active toward the CO oxidation. Given that we found similar oxygen step species in these kinked surfaces, our results predict a higher activity for the CO oxidation at oxygen-covered Pt kinked planes compared to that in the flat (111) plane.

## Conclusions

The CO and O_2_ adsorption on kinked Pt(111) vicinals has been studied using a curved crystal surface with the Pt(645) plane (α = 9.3°, 5-atom-wide terraces) in the center of the sample, allowing probe of vicinal surfaces beyond the densely packed Pt(312) facet (α = 22.2°, 2-atom-wide terraces). The curved surface allows for a systematic analysis of the variety of species observed at steps and terraces upon the separate adsorption of CO and O_2_. We demonstrate that CO predominately adsorbs at the kinked edges at low coverages, and only after the steps are near saturation, the adsorption at the terraces begins. The contrary was observed during desorption experiments, which reveals a higher adsorption energy of CO at kinks as compared to (111) terraces. In addition, our data shows how CO fully saturates the kinked edges with roughly one molecule per step atom, which may turn into a strong poisoning of the steps during CO oxidation conditions. O_2_ adsorption experiments reveal a significantly higher oxygen coverage at vicinal surfaces with kinked steps as compared to Pt(111) terraces. This effect is partly due to step-edge roughening and partly due to the presence of three additional step-like oxygen species, i.e., O chemisorbed at bridge-like Pt sites of the {100} microfacet and *fcc* positions of the {111} microfacet at the kinks, and oxygen forming 1D oxide chains, which grow also along the steps. The higher coverage and adsorption energy of CO at kinks point toward a higher ignition temperature, while the larger O coverage at kinks may indicate an increased reactivity under CO oxidation conditions.

## Supporting Information Available

LEED and Pt 4*f* core level of the clean and CO-saturated (111), (645), and (312) Pt surfaces; C 1s and O 1s evolution during CO desorption experiments after surface saturation with 10 L CO at the same surfaces; O 1s α-scans at 90 K after a 0.25 and 10 L CO dose, and at 300 K after 50 L O_2_ dose; and additional details on the W-model (PDF)
