# Supplementary material for: CO and O2 Interaction with Kinked Pt Surfaces
Source: ACS Catal. 2024 Apr 10;14(8):6319–27. doi: 10.1021/acscatal.4c00435 (PMC11037391; doi:10.1021/acscatal.4c00435)
Supplement: Supplementary file 1 — cs4c00435_si_001.pdf [file cs4c00435_si_001.pdf]

Supplementary information: CO and O_2 interaction with kinked Pt surfaces

Fernando García-Martínez,[*,†,§] Elia Turco,[*,†,‖] Frederik Schiller,[*,†] and J. Enrique Ortega[*,†,‡,¶]

†*Centro de Física de Materiales CSIC/UPV-EHU-Materials Physics Center, Manuel Lardizábal 5, San Sebastián 20018, Spain*

‡*Departamento Física Aplicada, Universidad del País Vasco, 20018 San Sebastián, Spain*

¶*Donostia International Physics Centre, Manuel Lardizábal 4, San Sebastián 20018, Spain*

§*Current address: Deutsches Elektronen-Synchrotron DESY, Notkestraße 85, Hamburg, 22607,Germany*

‖*Current address: Nanotech@surfaces Laboratory, Empa - Swiss Federal Laboratories for Materials Science and Technology, Ueberlandstrasse 129, Dübendorf, 8600, Switzerland*

E-mail: fernando.garcia-martinez@desy.de; elia.turco@empa.ch; frederikmichael.schiller@ehu.eus; enrique.ortega@ehu.eus

LEED and Pt 4f core levels

Low-Energy Electron Diffraction (LEED) patterns of the curved sample were used to check the sample surface orientation and superstructure. The characteristic hexagonal diffraction pattern corresponding to the (111) *fcc* terraces was found close to the border of the crystal edge. Away from that point, the LEED pattern reveals a steady split of the LEED spots. This splitting results from the additional superstructure related to the ordered steps of the surface.[1] The splitting increases with the step density due to the narrowing of the terraces (and a shorter real lattice constant),[2–4] becoming very pronounced at the highly stepped (312) plane. In the Pt $4f$ region, peaks related to bulk and terrace Pt are easily detected in the (111) surface. At the kinked planes, the terrace contribution decreases in favor of step Pt atoms.[3]

We also checked the LEED after CO saturation. A sharp $c(4 \times 2)$ diffraction pattern was observed at the (111) plane, rapidly vanishing after electron beam exposure, revealing a strong sensitivity to the low-energy electron beam of the LEED apparatus. The kinked surfaces only featured the characteristic splitting of spots due to the increasing step density, hence we conclude that CO molecules do not arrange in a long-range ordered manner at the kinked vicinals and there is no step doubling. However, since the $c(4 \times 2)$ LEED pattern rapidly vanished at the (111) plane, another possibility is that we are disordering the adsorbate structure during the LEED measurement itself. The Pt $4f$ spectrum at the (111) plane features, in addition to terrace and bulk Pt atoms, peaks corresponding to CO adsorbed in top and bridge sites. Similarly as before, at the stepped surfaces a contribution from CO adsorbed at steps is also observed, while those of terrace species decrease.

For O_2, the $p(2\times2)$ is neatly observed in the (111) plane, fading rapidly away as we move across the curved surface. In this case, the step splitting of the Pt vicinal substrate also vanishes, indicating that O adsorption causes a structural disruption of the step lattice, reflecting its higher interaction with the substrate. We acquired no Pt $4f$ spectra in this case.

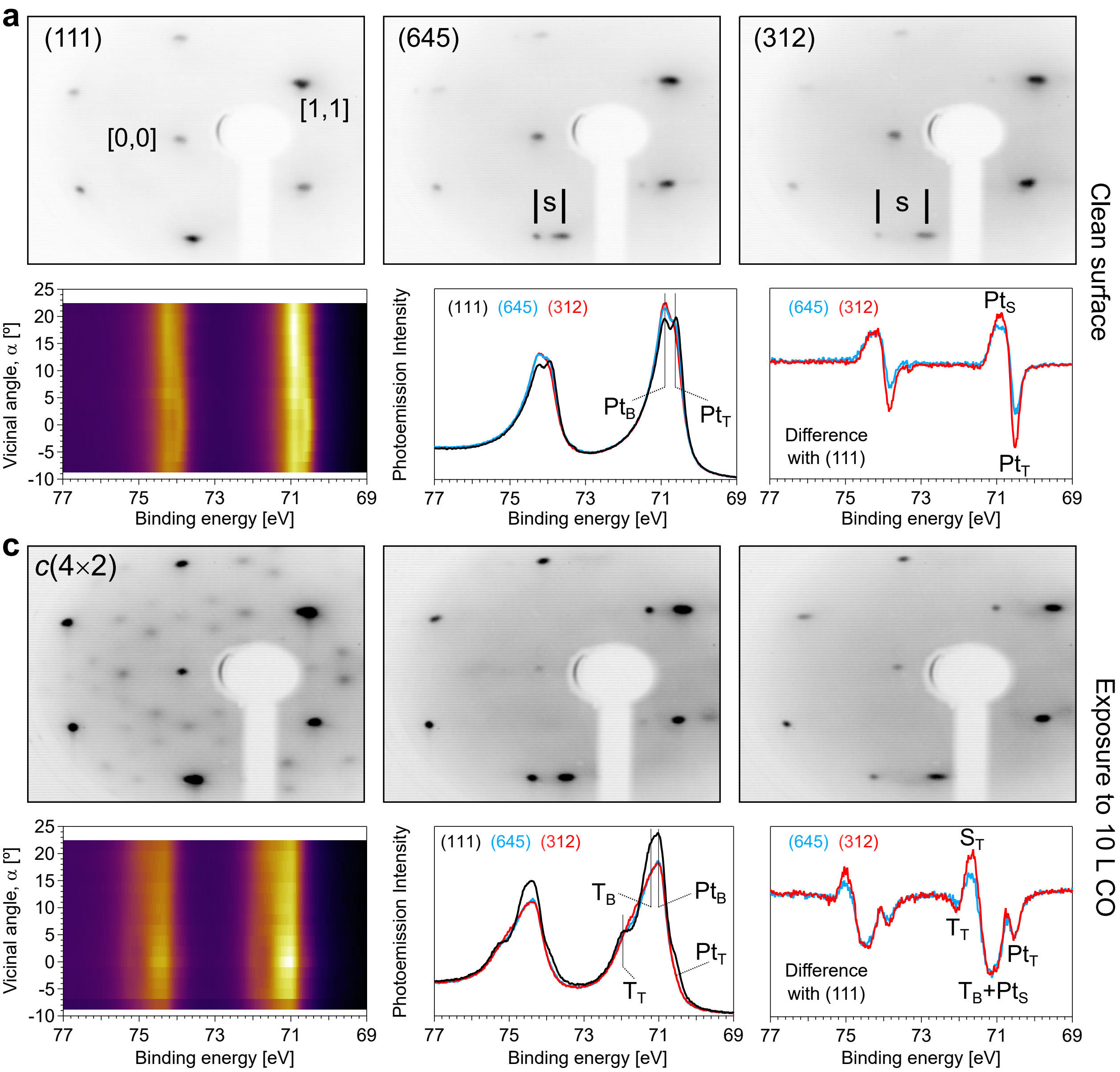

Figure S1: **LEED patterns and Pt 4*f* region of the clean and CO-covered surfaces.** LEED diffraction images and Pt 4*f* XPS regions obtained after cleaning the sample (**a**, 300 K) and after dosing 10 L CO (**c**, dose at 300 K, measurement at 90 K) at the (111), (645) and (312) planes, respectively. In the LEED images, the increasing splitting distance of the spots as α increases is labelled as **s**.[4,5] Positions of the [0,0] and [1,1] spots are also indicated. In the Pt 4*f* spectra, Pt_B, Pt_T and Pt_S refer to bulk, terrace and step Pt atoms, while T_T, T_B and S_T belong to CO molecules anchored in Terrace-Top, Terrace-Bridge and Step-Top sites. Differences at the stepped surfaces are better appreciated when subtracting the corresponding spectra at the (111) plane. As expected, terrace components (Pt_T, T_T, T_B) decrease at the stepped surfaces, in parallel to the growth of step-related peaks (Pt_S, S_T).

C 1s evolution during the CO desorption

The desorption of CO is the fundamental process that triggers the catalytic CO oxidation. We investigate such phenomenon in Fig. S2 and S3. To this aim, the (111), (645) and (312) surfaces were CO-saturated at 300K with a 10 L dose, and subsequently heated at a constant heating rate of 5 K/min while continuously recording XPS. A photon energy of 650 eV was chosen to also probe the O 1s region. As in previous discussions, the well-documented (111) plane was used as a reference (left column of Fig. S2).[6–9] As seen in Fig. S2**d,g**, T_B starts to desorb right after starting the heating ramp, and vanishes at around 390 K. However, T_T remains rather stable until 320 K, then it starts to decrease, and completely desorbs at around 430 K, i.e., approximately 40 K higher than T_B. Earlier desorption of CO anchored at T_B positions rather than at T_T sites is indeed expected.[6,8,10]

At the (645) surface (center column of Fig. S2), as in the (111) plane, the first species to desorb from the CO-covered surface is T_B-CO. It completely vanishes at 360 K, followed by T_T-CO at around 400 K, and by S_T-CO at 475K, illustrating the larger adsorption energy of CO adsorbed at steps as compared to its terrace analogous.[11–13] The evolution is similar in the CO-saturated (312) surface (right column of Fig. S2), where the main contribution is S_T-CO, while the peak related to CO adsorbed at T_T sites is remarkably smaller. In the (312) surface there is an additional feature initially attributed to CO anchored at T_B sites, slightly shifted as compared to the (111) plane (285.75 and 285.93 eV, respectively), which was also observed during the uptake experiments. Nevertheless, upon heating the T_T-CO peak starts to decrease prior to this 285.75 eV feature. Additionally, this latter contribution remains almost constant with temperature and abruptly vanishes at 390 K. This reveals differences with CO molecules adsorbed at T_B sites on large (111) terraces, as the latter would readily start to desorb rapidly after increasing the temperature above room temperature.[14] The peak at 285.75 eV is that related to CO adsorbed at defects,[11] which can only be resolved in the (312) surface at this temperature (it is resolved in all surfaces at 90 K, see Fig. 3 in the text). CO anchored at both T_T and defect sites completely desorb at 390 K. S_T does

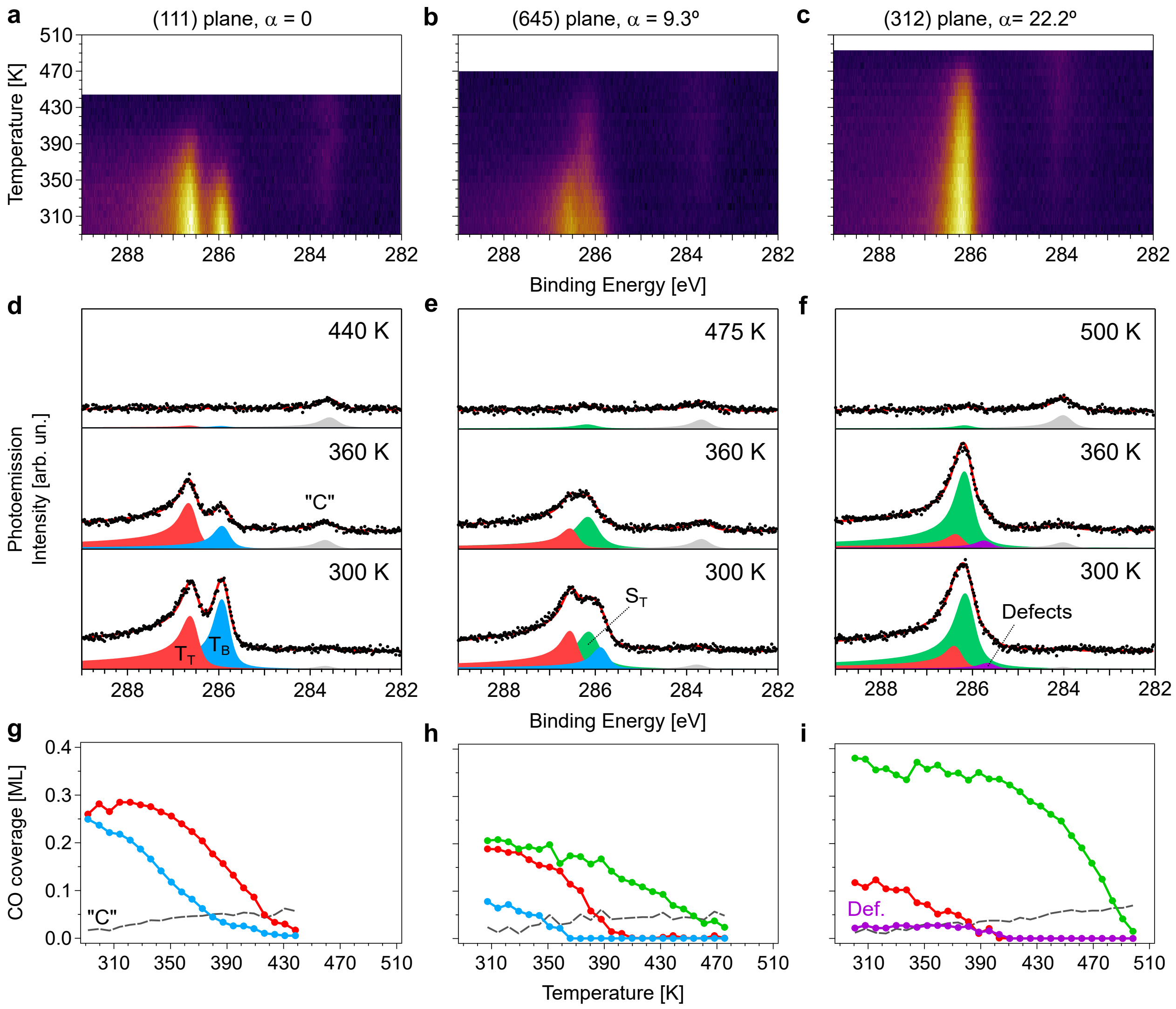

Figure S2: **C 1s evolution during CO desorption experiments. a-c** Photoemission intensity plots of the C 1s region during separate heating ramps in CO-saturated (111), (645) and (312) surfaces, respectively. The surfaces were exposed to 10 L CO in order to ensure saturation. **d-f** Selected fitted spectra at the (111), (645) and (312) planes at the beginning (300 K), middle (360 K) and end (440, 475 and 500 K, respectively) of each desorption experiment. **g-i** Coverage evolution as a function of temperature of individual CO species, extracted from the fit of all spectra shown in a-c). CO adsorption as Terrace-Top, Terrace-Bridge and Step-Top are denoted as T_T, T_B and S_T, while graphitic carbon is denoted as "C". CO chemisorbed at defect sites at the (312) surface is colored in purple. CO adsorption at defects of the (312) surface is colored in purple. The experiments were carried out at a photon energy of 650 eV to also monitor the O 1s region, shown in the SI.

so at 500 K, reflecting again a larger desorption temperature of CO adsorbed at steps as compared to terraces.

The three desorption experiments confirm the expected trend for desorption temperatures: $T_B \rightarrow T_T \rightarrow S_T$, *i.e.*, the reverse sequence with respect to the one observed during the uptakes discussed along with Fig. 2 of the main text. Impinging CO molecules adsorb first on kinks in the low coverage regime, and they will desorb from them only after the CO from the terraces has almost vanished. Accordingly, the bigger desorption temperature of CO adsorbed at steps is of relevance to the CO oxidation ignition in NAP experiments on these vicinal surfaces. Regarding graphitic carbon "C", it increases during the three desorption experiments. We also found that CO anchored at T_B sites dissociates under more intense photon beams, pointing to beam-damage rather than to heating-induced CO cracking.

O 1*s* evolution during the CO desorption

The O 1*s* region was also recorded during the CO desorption experiments as shown in Fig. S3. No oxygen arising from the CO dissociation was detected in the O 1*s* region in any of the studied surfaces. We observed pronounced shifts at the (645) and (312) stepped surfaces as CO desorbs, caused by an earlier desorption of CO anchored at T_T sites (532.6 eV) prior to S_T positions desorption (532.3 eV).[11] No shift was observed for T_B.

Another aim of this experiment was to monitor the peak position of T_T-CO in the (111) surface during the CO desorption, since a significant shift was observed during the CO ignition at high pressures on Pt(111) (see[15]). No shift of T_T-CO was found at the (111) plane, indicating that the shift observed during the CO oxidation experiment is not directly related to the CO desorption process.

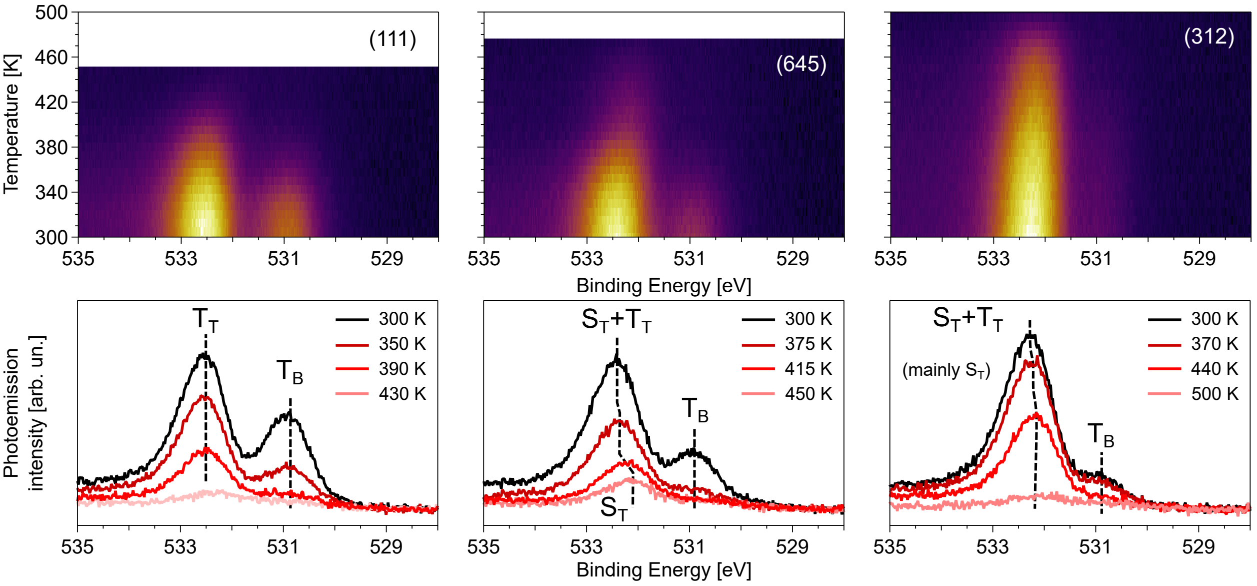

Figure S3: **O 1*s* evolution during CO desorption cycles.**
O 1*s* temperature ramps during the CO desorption experiments of Fig. S2 are shown in the top row. Individual spectrum at different temperatures appear in the bottom. Dashed black lines indicate the approximate position of peak maxima in order to illustrate the shifts of the peaks. Experiments were carried out at a photon energy of 650 eV and a heating slope of 5 K/min.

O 1s α-scans at low and high CO dose

The O 1s region was scanned at 650 eV after the exposure to 0.25 and 10 L CO at 300 K (Fig. S4). Contrary to the C 1s region, peaks from CO anchored at T_T (532.6 eV) and S_T (532.3 eV) sites are not resolved in the O 1s.[11] In addition, it is clear that the ratio between CO adsorbed at T_B (531.0 eV) and T_T sites (532.5 eV) is not 1, reflecting that the O 1s spectrum at the (111) plane suffers from PED effects.[16] A closer look to the α-scans reveals a significant binding energy shift with α in the peak around 532.5 eV. This is expected, since at low α, the main contribution is T_T-CO (532.6 eV), yet at the densely stepped surfaces S_T-CO (532.3 eV) is the main species.

A substantial increment in the total intensity with α was observed. This effect is also attributed to enhanced O 1s emission due to PED effects and the curved shape of the crystals, and not to such large increase in the total coverage. The same intensity growth with α after CO saturation was observed for close-packed Pt vicinals, as described in Ref.[5]

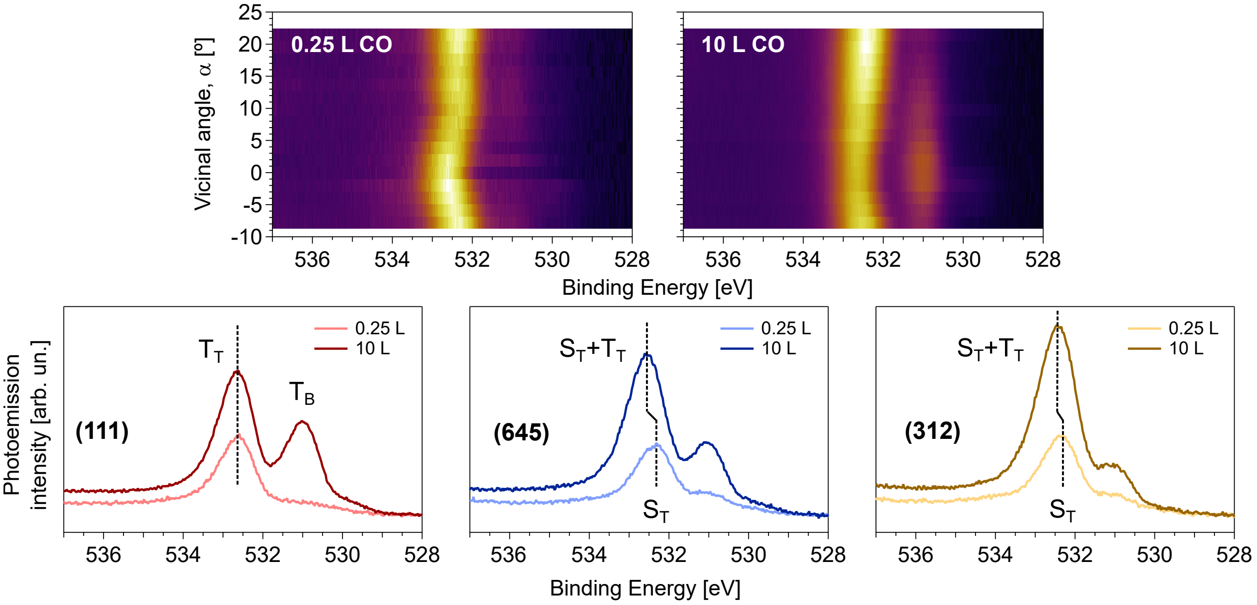

Figure S4: **O 1s α-scans for the 0.25 and 10 L CO dose.**
The O 1s α-scans at 0.25 and 10 L CO doses are shown in the top row, together with spectrum for the (111), (645) and (312) planes in the bottom. Following the previous discussion, CO adsorbed as Terrace-Top, Terrace-Bridge and Step-Top are denoted by T_T, T_B and S_T, respectively. The surface was exposed to CO at 300 K, although the spectra were measured at 90 K to improve thermal resolution. The photon energy was 650 eV.

Additional details on the *W*-model

The so-called W-model was introduced in Ref.,[5] aiming at fitting the linear variation of the coverage of adsorbates anchored at terraces and steps as a function of α, which in turn enables to estimate the lateral size of the steps W, *i.e.* their region of influence at neighbouring terraces. In the present work, we start by fitting Terrace-CO variation of Fig. 3**f** as a function of α [$\Theta_T(\alpha)$] to Eq. 1 below, resulting in the dotted red line in Fig. 3**f**.

$$\Theta_T(\alpha) = \Theta_T^0[1 - W \cdot \frac{\sin(|\alpha|)}{h}] \tag{1}$$

In this equation, Θ_T^0 corresponds to the coverage at the flat (111) surface, which in the present case is the 0.5 ML saturation, and $h = 2.26$ Å refers to the height of a Pt step.[5] The fit returns $W = 4.3 \pm 0.1$ Å, which corresponds to about 1.55 times the distance between consecutive Pt rows in the direction perpendicular to the kinks (2.77 Å), suggesting that the influence of the step extends by a 55% of an atomic row inside the lower terrace. Both magnitudes are sketched in Fig. 3**g**. Using $W = 4.3$ Å as input parameter, one can now fit the Step-CO variation as a function of α [$\Theta_S(\alpha)$] with the following equation:

$$\Theta_S(\alpha) = \Theta_S^0 \cdot W \cdot \frac{\sin(|\alpha|)}{h} \tag{2}$$

This fit renders $\Theta_S^0 = 0.71 \pm 0.02$ ML, which accounts for the local coverage within the W_K-region. One may also define $a_\perp$ as the distance between rows in the direction perpendicular to the kinked steps, which results into $a_\perp = a_0\frac{\sqrt{2}}{2} = 2.77$, being a_0 the lattice parameter of Pt(3.92 Å). Therefore, by doing $\Theta_S^0 \cdot W/a_\perp$ we obtain the number of adsorbed molecules per kink row, yielding 1.1 ± 0.05 CO molecules per kink atom, i.e., full CO occupation of the kinked edge at all vicinal angles.

O 1s α-scan at O saturation at 300K

The O 1s α-scan after O_2 saturation at 300 K is shown in Fig. S5. To avoid the reduction of the O species by residual CO, we limited the α-scan to only 8 points. Nevertheless, the tendency is the same: the O coverage increases dramatically at kinked surfaces, immediately away from the (111) plane. The chemical species found are the same as in the α-scan at 90 K, although the total coverage is smaller. As mentioned in the main text, we attribute this deviation in total coverage to partial surface reduction, caused by the residual CO in the chamber.

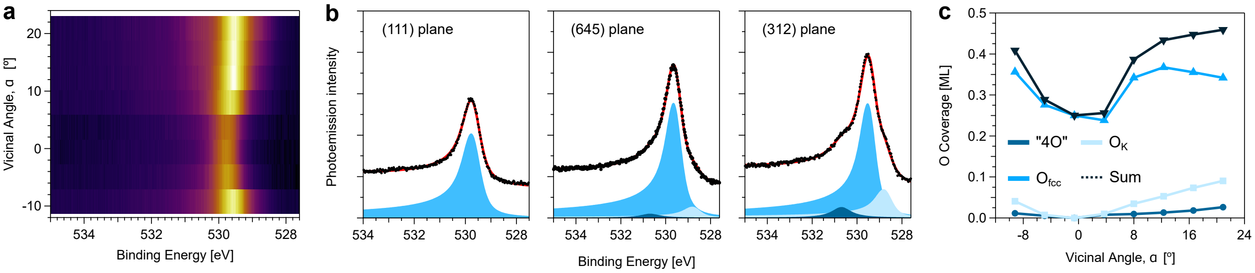

Figure S5: **Surface scan for O-saturated sample at 300 K.**
a O 1s α-scans after exposure to 50 L O_2 at 300 K. **b** Individual O 1s photoemission spectra for the (111), (645) and (312) planes. O_K and O_{fcc} allude to chemisorbed O at square sites at the kinks and at hollow *fcc* sites at steps and terraces, while 4O stands for 1D chains of the "4O" oxide. Refer to Fig. 4 for more details about the nature of these chemical species. Experiments carried out at a photon energy of 650 eV.

References

(1) Comsa, G.; Mechtersheimer, G.; Poelsema, B. He-beam scattering study of the dynamics of oxygen induced reconstruction of the Pt(997) surface. *Surface Science* **1982**, *119*, 159–171.

(2) Ortega, J. E.; Corso, M.; Abd-el Fattah, Z. M.; Goiri, E. A.; Schiller, F. Interplay between structure and electronic states in step arrays explored with curved surfaces. *Phys. Rev. B* **2011**, *83*, 085411.

(3) Walter, A. L.; Schiller, F.; Corso, M.; Merte, L. R.; Bertram, F.; Lobo-Checa, J.; Shipilin, M.; Gustafson, J.; Lundgren, E.; Brión-Ríos, A. X.; Cabrera-Sanfelix, P.; Sánchez-Portal, D.; Ortega, J. E. X-ray photoemission analysis of clean and carbon monoxide-chemisorbed platinum(111) stepped surfaces using a curved crystal. *Nature Communications* **2015**, *6*, 8903.

(4) Ilyn, M.; Magaña, A.; Walter, A. L.; Lobo-Checa, J.; De Oteyza, D. G.; Schiller, F.; Ortega, J. E. Step-doubling at Vicinal Ni(111) Surfaces Investigated with a Curved Crystal. *Journal of Physical Chemistry C* **2017**, *121*, 3880–3886.

(5) Garcia-Martinez, F.; Schiller, F.; Blomberg, S.; Shipilin, M.; Merte, L. R.; Gustafson, J.; Lundgren, E.; Ortega, J. E. CO chemisorption on vicinal Rh(111) surfaces studied with a curved crystal. *The Journal of Physical Chemistry C* **2020**, *124*, 9305–9313.

(6) Froitzheim, H.; Schulze, M. The kinetics of the adsorption and desorption of the system CO/Pt(111) derived from high resolution TREELS. *Surface Science* **1989**, *211-212*, 837–843.

(7) Hopster, H.; Ibach, H. Adsorption of CO on Pt(111) and Pt 6(111) $\times$ (111) studied by high resolution electron energy loss spectroscopy and thermal desorption spectroscopy. *Surface Science* **1978**, *77*, 109–117.

(8) Gland, J. L.; Kollin, E. B. Vibrational characterization of carbon monoxide oxidation on the Pt(111) surface. *Surface Science* **1985**, *151*, 260–270.

(9) Campbell, C.; Ertl, G.; Kuipers, H.; Segner, J. A molecular beam investigation of the interactions of CO with a Pt(111) surface. *Surface Science* **1981**, *107*, 207–219.

(10) Campbell, C.; Ertl, G.; Kuipers, H.; Segner, J. A molecular beam study of the adsorption and desorption of oxygen from a Pt(111) surface. *Surface Science* **1981**, *107*, 220–236.

(11) Tränkenschuh, B.; Fritsche, N.; Fuhrmann, T.; Papp, C.; Zhu, J. F.; Denecke, R.; Steinrück, H. P. A site-selective in situ study of CO adsorption and desorption on Pt(355). *Journal of Chemical Physics* **2006**, *124*, 1–10.

(12) Xu, J.; Yates, J. T. Terrace width effect on adsorbate vibrations: a comparison of Pt(335) and Pt(112) for chemisorption of CO. *Surface Science* **1995**, *327*, 193–201.

(13) Tränkenschuh, B.; Papp, C.; Fuhrmann, T.; Denecke, R.; Steinrück, H. P. The dissimilar twins - a comparative, site-selective in situ study of CO adsorption and desorption on Pt(322) and Pt(355). *Surface Science* **2007**, *601*, 1108–1117.

(14) Kinne, M.; Fuhrmann, T.; Whelan, C. M.; Zhu, J. F.; Pantförder, J.; Probst, M.; Held, G.; Denecke, R.; Steinrück, H. P. Kinetic parameters of CO adsorbed on Pt(111) studied by in situ high resolution x-ray photoelectron spectroscopy. *Journal of Chemical Physics* **2002**, *117*, 10852–10859.

(15) Garcia-Martinez, F.; García-Fernández, C.; Simonovis, J. P.; Hunt, A.; Walter, A.; Waluyo, I.; Bertram, F.; Merte, L. R.; Shipilin, M.; Pfaff, S.; Blomberg, S.; Zetterberg, J.; Gustafson, J.; Lundgren, E.; Sánchez-Portal, D.; Schiller, F.; Ortega, J. E. Catalytic Oxidation of CO on a Curved Pt(111) Surface: Simultaneous Ignition at All Facets through a Transient CO-O Complex**. *Angewandte Chemie International Edition* **2020**, *59*, 20037–20043.

(16) Toyoshima, R.; Yoshida, M.; Monya, Y.; Suzuki, K.; Amemiya, K.; Mase, K.; Mun, B. S.; Kondoh, H. A high-pressure-induced dense CO overlayer on a Pt(111) surface: A chemical analysis using in situ near ambient pressure XPS. *Physical Chemistry Chemical Physics* **2014**, *16*, 23564–23567.
